# Pharmacological Inhibition of TPL2/MAP3K8 Blocks Human Cytotoxic T Lymphocyte Effector Functions

**DOI:** 10.1371/journal.pone.0092187

**Published:** 2014-03-18

**Authors:** Fatema Z. Chowdhury, Leonardo D. Estrada, Sean Murray, James Forman, J. David Farrar

**Affiliations:** 1 Department of Immunology, The University of Texas Southwestern Medical Center, Dallas, Texas, United States of America; 2 Department of Molecular Biology, The University of Texas Southwestern Medical Center, Dallas, Texas, United States of America; University of Iowa, United States of America

## Abstract

CD8^+^ cytotoxic T lymphocytes (CTLs) play a major role in defense against intracellular pathogens. During development, antigen-presenting cells secrete innate cytokines such as IL-12 and IFN-α, which drive CTL differentiation into diverse populations of effector and long-lived memory cells. Using whole transcriptome analyses, the serine/threonine protein kinase Tpl2/MAP3K8 was found to be induced by IL-12 and selectively expressed by effector memory (T_EM_) CTLs. Tpl2 regulates various inflammatory pathways by activating the ERK mediated MAP kinase pathway in innate immune cells such as macrophages and dendritic cells. In this study, we found that a specific small molecule Tpl2 inhibitor blocked IFN-γ and TNF-α secretion as well as cytolytic activity of human CTLs. This pathway was specific for human effector CTLs, as the Tpl2 inhibitor did not block IFN-γ and TNF-α secretion from murine effector CTLs. Further, IL-12 failed to induce expression of Tpl2 in murine CTLs, and Tpl2 deficient murine CTLs did not exhibit any functional deficiency either *in vitro* or *in vivo* in response to *L. monocytogenes* infection. In summary, we identified a species-specific role for Tpl2 in effector function of human CTLs, which plays a major role in adaptive immune responses to intracellular pathogens and tumors.

## Introduction

CD8^+^ cytotoxic T lymphocytes (CTLs) regulate immunity to intracellular infections and tumors by secreting pro-inflammatory cytokines and killing infected cells. These functions are acquired by naïve CTLs during their initial priming in response to both antigen recognition and innate cytokines [1]. IL-12 and IFN-α/β are potent “signal 3” inflammatory cytokines that are involved in activation and programming of naïve CD8^+^ T cells (T_N_) in mice [2]–[5]. Once infection has subsided, cell death occurs in the majority of antigen-specific CTLs, and a small subset of memory CTLs persist with the ability to respond more rapidly and robustly upon reinfection with the same pathogen. There are two types of memory CTL that persist *in vivo*. While central memory CD8^+^ T cells (T_CM_) require cell division to give rise to effector CTLs, the effector memory CD8^+^ T cells (T_EM_) are capable of immediate effector function upon antigen presentation without the need for additional signals [6]. We have previously demonstrated that IL-12, but not IFN-α, programs effector function in human CD8^+^ T cells [7], [8]. We have determined that IL-12 regulates a distinct set of genes involved in effector function. Moreover, some of the IL-12 regulated genes were also stably expressed within the T_EM_ CTLs *ex vivo* when compared to the T_N+CM_ CTLs [8]. Within this gene signature we identified a MAP kinase pathway intermediate, Tpl2, to be differentially regulated by IL-12. The goal of this study is to test the role of Tpl2 in effector function of human CD8^+^ T cells.

MAP3K8 or Tpl2 (also known as Cot or c-Cot) is a serine-threonine protein kinase and belongs to the MAPKKK family [9]. Tpl2 was first discovered in the early 1990s as a proto-oncogene [10]–[12]. DNA isolated from a specific human thyroid carcinoma cell line showed the ability to transform the hamster embryonic cell line (SHOK) *in vitro*, which was the first description of the human Cot (cancer Osaka thyroid) oncogene [10]. The rat homolog of Cot, Tpl2 (tumor progression locus-2) was then identified and found to transform NIH 3T3 fibroblasts *in vitro* [11]. LPS, TNF-α, and IL-1β can activate Tpl2 in the innate immune cells. Macrophages from Tpl2 KO mice exhibit a defect in ERK phosphorylation as well as TNF-α secretion in response to LPS stimulation [13], [14]. While Tpl2 can directly phosphorylate MEK1/2, leading to ERK phosphorylation, depending upon the cell type and stimulus, it can be dispensable for p38 and JNK activation in innate immune cells in mice [9], [12]. In CD4^+^ T cells, Tpl2-dependent activation of T helper 1 cells is required for optimal IFN-γ production and subsequent clearance of *Toxoplasma gondii* in mice [15]. ERK activation has been shown to be important for CD8^+^ T cell development in the thymus [16], [17]. Total T cell metabolism as well as CTL proliferation and survival have been linked to ERK activation as well [18], [19]. However, the role of Tpl2 in triggering ERK pathway in the activation of CTL effector functions is still unknown.

In this study, we addressed the role of Tpl2 in regulating both mouse and human CTL effector function. While human CTLs show marked inhibition of cytokine secretion and lytic activity in the presence of a small molecule inhibitor of Tpl2, we found no requirement for this pathway in mouse CD8^+^ T cells. Thus, our findings highlight the unique and species-specific role that Tpl2 plays in human CTL effector functions.

## Materials and Methods

### Human subjects and ethics statement

Peripheral blood (120–180cc) was collected by venipuncture from healthy adult donors. Written informed consent was obtained from each donor in the presence of study personnel and a witness. This study, the informed consent process, and all documentation were approved by the Internal Review Board at the University of Texas Southwestern Medical Center. Informed consent was obtained in accordance with the Declaration of Helsinki.

### Mice, ethics statement, and *Listeria monocytogenes* infections

All experiments and procedures involving animals described in this study were specifically approved by the Institutional Animal Care and Use Committee at UTSW Medical Center. All mice were housed in specific pathogen-free facilities. The generation of Tpl2^−/−^ mice was described previously, and these mice were a kind gift from Dr. Philip Tsichlis (Tufts University) [13]. For primary infections, mice received 2,000 CFU/mouse *L. monocytogenes* expressing ovalbumin (LM-OVA) via i.v. injection, and 20,000 CFU/mouse for secondary infection. CFU counts for injected bacteria were confirmed by colony count from bacterial growth on BHI agar plates. Blood was collected retro-orbitally to confirm primary expansion of CD8^+^ T cells when a secondary infection was also performed. Spleens and lymph nodes were harvested and splenocytes were isolated for further analyses.

### Isolation and culture of CD8^+^ T cells

Human peripheral blood mononuclear cells (PBMCs) were isolated from whole blood by ficoll density centrifugation, and CD8^+^CD45RA^+^ cells were isolated by negative selection with BD IMag kit (Human Naïve CD8^+^ T Cell Enrichment Set – DM). Purity was routinely greater than 90%. Purified CD8^+^CD45RA^+^ cells were cultured at 1e^6^ cells/mL in 96-well tissue culture treated plates coated with 1.5 μg/mL of anti-CD3+anti-CD28 antibodies in complete IMDM supplemented with 10% FBS. Cells were cultured with rhIL-2 (200 U/ml) and anti-human IFN-γ (5 μg/mL) with the following specific conditions: “Neutralized” (anti-human IL-12 [5 μg/mL]), “IL-12” (rhIL-12 [5 ng/mL]), “IFN-α(anti-human IL-12 [5 μg/mL],+ recombinant-human IFN-α(A) [1000 U/mL]), and “IL-12+IFN-α”rhIL-12 [10 ng/mL], + recombinant-human IFN-α(A) [1000 U/mL]). Unless otherwise noted, cells were split 1:10 with additional 100 U/mL rhIL-2. Also, 200 U/mL rhIL-2 was added during secondary stimulation unless otherwise specified in the figure legends. All secondary stimulation for ELISA were performed in the presence of plate-bound anti-CD3 stimulation only.

Murine CTLs were stimulated *in vitro* with plate-bound anti-CD3 (Clone 2C11) and anti-CD28 (Clone 37.51). For optimal primary stimulation, 0.5 μg/mL of each antibody was used to coat tissue culture treated plates prior to stimulation. Cells were cultured under neutralized (anti-mouse IL-4 + anti-mouse IFN-γ+ anti-mouse IL-12 + rhIL-2 (cross-reactive with mouse) [200 U/mL]), IL-12 (anti-mouse IL-4 + anti-mouse IFN-γ+ rmIL-12 [10 ng/mL] + rhIL-2 [200 U/mL]), or IFN-α(anti-mouse IL-4 + anti-mouse IFN-γ+ anti-mouse IL-12 + rhIFN-α (universal Type I, A/D) [1000 U/mL] + rhIL-2 [200 U/mL]) conditions.

### Intracellular cytokine staining and flow cytometry

For intracellular cytokine staining, surface staining was performed prior to fixing using 5% formaldehyde in 1X PBS. Cells were then washed in 1X PBS alone and permeabilized with buffer containing 0.1% saponin in 1X PBS+0.5% BSA. Cells were stained with the following antibodies diluted in the permeabilizing buffer: anti-mouse IFN-γ conjugated to PerCP-Cy5.5 (Biolegend, XMG1.2, 505822) and anti-mouse TNF-α conjugated to FITC (Biolegend, MP6-XT22, 506304). All data were collected using FACSAria flow cytometer and analyzed using FlowJo software (TreeStar).

### Western blot analysis and infrared imaging

Cell lysates were prepared by incubating cells (5×10^7^ cells/group) for 1 hour at 4°C in radioimmune precipitation assay (RIPA) buffer (50 mM Tris-HCl, pH 8.0, 150 mM NaCl, 0.1% sodium dodecyl sulfate (SDS), 0.5% sodium deoxycholate, 1% Tween-20) with proteinase and phosphatase inhibitors (1 μM phenylmethylsulfonyl fluoride (PMSF), 1 μM dithiothreitol (DTT), 10 μg/ml leupeptin, 1 μM benzamidine, 1 μM pepstatin, and 1 μM Na_3_VO_4_). Protein (20 μg) was resolved by SDS-PAGE and transferred to polyvinylidene difluoride (PVDF) membranes and blotted with rabbit-anti-MAP3K8/Tpl2 (Santa Cruz, sc-720), rabbit-anti-phophoERK (Cell Signal, 9101), or with rabbit-anti-ERK (Cell Signal, 9102). Mouse monoclonal antibody against GAPDH was used as loading control (Sigma, G8795). For Tpl2 detection, goat anti-rabbit conjugated to IRDye 800CW and Goat anti-mouse conjugated to IRDye 680 secondary antibodies (Gift from Li-Cor Biosciences; Cat# 827-08365 and 827-08366, respectively) were used to detect primary antibody. Infrared signal was detected by scanning the membrane with Odyssey Infrared Imaging System (Li-Cor Biosciences). For ERK and p-ERK detection, membranes were blotted with donkey-anti-rabbit-HRP secondary antibody and detected with chemilluminescence detection reagent (Amersham).

### Kinase inhibitors

Tpl2 Kinase Inhibitor (Santa Cruz, sc-204351), U-0126 (Santa Cruz, sc-222395), and SB 203580 (Calbiochem, 559389) were used in this study. All kinase inhibitors were dissolved in 100% DMSO, and DMSO was used as the vehicle control treatment for all experiments.

### Re-directed lysis (cytotoxicity) assay

CD8^+^ cells were sorted by FACS based on chemokine receptors and were subjected to a redirected lysis assay as previously described [8]. Briefly, anti-CD3-coated THP-1 target cells were labeled by culturing in the presence of 150 μCi Na_2_[^51^Cr]O_4_ in complete growth media for 1.5 h. Target cells were washed and incubated with CTLs at various effector:target (E:T) ratios for 4 h at 37°C. Specific cytotoxicity was measured by scintillation counting of ^51^Cr released in the media.

### qPCR analysis

Total cellular RNA was isolated with RNeasy Mini Kit with DNaseI treatment (Qiagen) according to manufacturer's instructions. RNA was reverse transcribed with the ABI High Capacity cDNA Reverse Transcription Kit (Applied Biosystems). qRT-PCR was performed with Maxima SYBR Green qPCR Master Mix (Thermo Scientific), and reactions were quantified with the ABI7300 cycler (Applied Biosystems). Primers directed against PPIA were used as a reference for both human and murine gene expression. Relative expression of mRNA was calculated by the 2^−ΔΔCt^ method [20]. Primers sequences: hTpl2: For 5′-CAGTAATCAAAACGATGAGCGTTCTA-3′, Rev 5′-GAACATCGGAATCTATTTGGTAACGT-3′; hPPIA: For 5′-GCGTCTCCTTTGAGCTGTTTGC-3′, Rev 5′-ATGGACTTGCCACCAGTGCC-3′; mIFNG: For 5′-ACAATCAGGCCATCAGCAACAAC-3′, Rev 5′-CAGCGACTCCTTTTCCGCTTC-3′; mTpl2 For 5′-TCAGTCCCCAGAATGGCCGCT-3′, Rev 5′-AGAACAGACCCTCCCTCGCCG-3′; mPPIA: For 5′-TTATTCCAGGATTCATGTGCCAGGG-3′, Rev 5′-TCATGCCTTCTTTCACCTTCCCAA-3′.

### Statistical analyses

Depending upon the experimental design, statistical differences were determined by either a two-tailed Student's t-test for simple pair-wise comparisons between two groups, or a two-way ANOVA with a post-hoc Bonferoni test to determine pair-wise differences in experiments with more that two treatment groups. Groups were considered significantly different if p≤0.05.

## Results

### Elevated Tpl2 expression in human T_EM_ CTLs and regulation by IL-12

**Figure 1:**
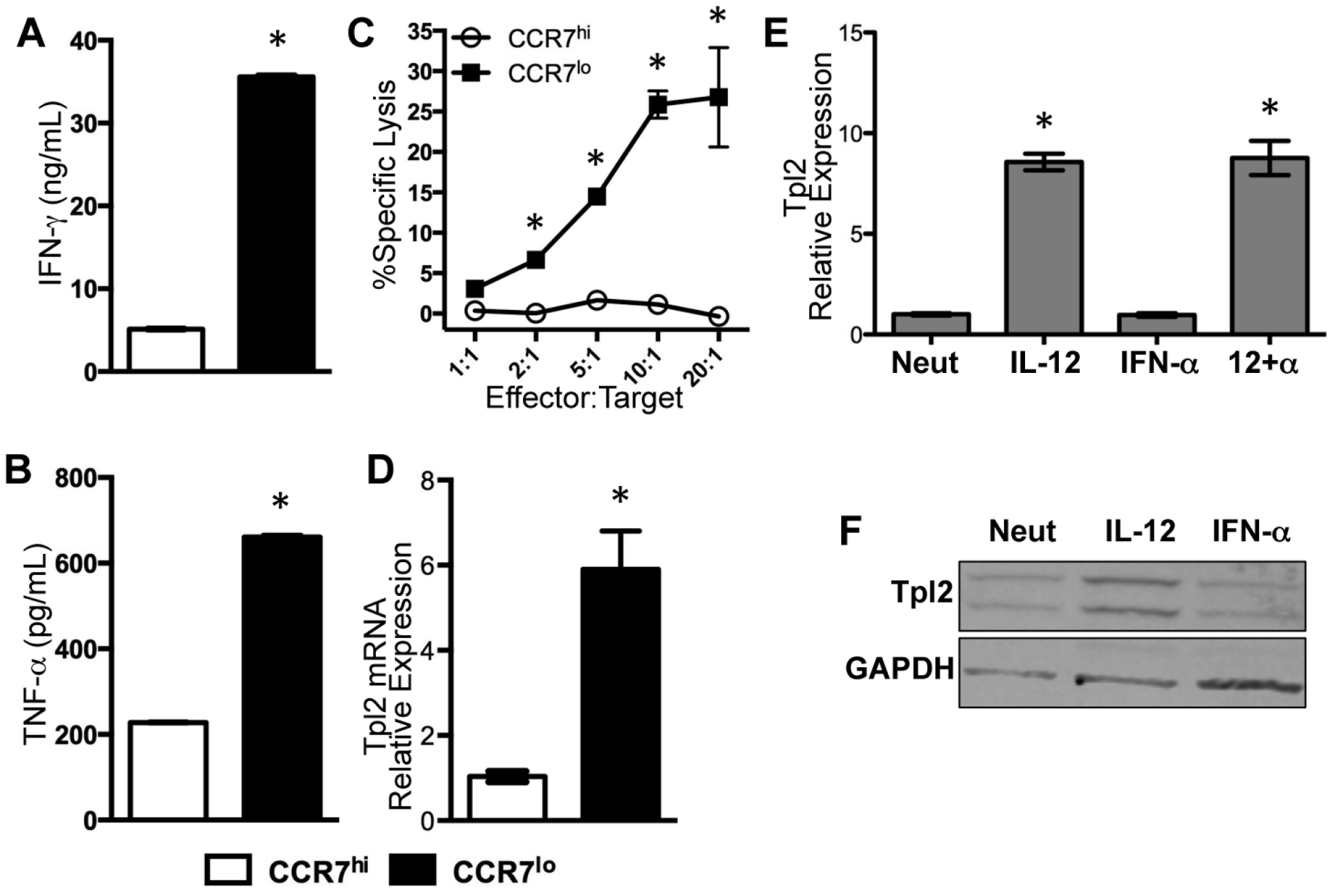
Tpl2 expression is elevated in human T_EM_ CTLs *ex vivo* and induced by IL-12 *in vitro*. (A-D) CCR7^l^°CD8^+^ and CCR7^hi^CD8^+^ T cells were sorted from healthy human PBMCs. (A, B) Sorted cells were stimulated *in vitro* with anti-CD3 and IL-2 (200 U/mL) for 24 h. (A) IFN-γ and (B) TNF-α in the supernatant were quantified by ELISA, and the results are representative of four individual experiments. (C) CCR7^l^°CD8^+^ and CCR7^hi^CD8^+^ sorted CTLs were were assessed for lytic activity in redirected lysis assay at the indicated Effector:Target ratios using anti-hCD3 coated THP-1 target cells. Percent specific lysis, mean±SD of technical replicates is plotted and is representative of two individual experiments. (D) Total RNA from CTLs was collected from *ex vivo* purified cells without stimulation. Tpl2 expression in CCR7^l^°CD8^+^ CTLs is plotted relative to CCR7^hi^CD8^+^ CTLs, and the experiment was performed twice with similar results. (E–F) Naïve CD8^+^ (CD8^+^CD45RA^+^) T cells were isolated from healthy human PBMCs and stimulated with plate-bound anti-CD3+anti-CD28 under neutralized (Neut), IL-12, IFN-α, or IL-12 + IFN-α (12+α) cytokine conditions. Total RNA or protein was collected 3.5 days post stimulation. (E) Tpl2 mRNA was measured by qRT-PCR and plotted (mean±SD) and data are expressed relative to the neutralized condition. Data are representative of three experiments. (F) Total protein (20 μg) of cell lysates was separated by SDS-PAGE and blotted for Tpl2 protein using rabbit polyclonal antibody against Cot/Tpl2. Alternative splicing results in two isoforms of the same protein and thus, two bands can be detected. GAPDH was measured as a loading control. Data are representative of four separate experiments. *, p≤0.05, and other pairwise comparisons were not significant.

We previously demonstrated that CD8^+^ CCR7^l^°CXCR3^hi^ T cells isolated from human peripheral blood contain T_EM_ CTLs that display robust effector function in the absence of additional cytokine stimuli [8]. CD8^+^CCR7^hi^ and CD8^+^CCR7^l^°CTLs were sort-purified from healthy human PBMCs (Figure 1A–D). CD8^+^CCR7^lo^ T cells secreted significantly more IFN-γ and TNF-α upon plate-bound anti-CD3 stimulation when compared to the CD8^+^CCR7^hi^ T cells (Figure 1A, B). CD8^+^CCR7^lo^ T cells also displayed immediate cytolytic activity while CD8^+^CCR7^hi^ CTLs lacked any significant killing of the target cells (Figure 1C). Although, the CD8^+^CCR7^lo^ T cells are a heterogeneous population, their immediate effector function indicated that it was likely comprised of T_EM_ CTLs when compared to CD8^+^CCR7^hi^ CTLs. In agreement with our previous microarray analysis [8], Tpl2 mRNA expression was indeed higher in the CD8^+^CCR7^lo^ population when compared with the CD8^+^CCR7^hi^ cells *ex vivo* without any stimulation as assessed by qPCR (Figure 1D). Thus, Tpl2 mRNA was more highly expressed in a cell population containing human T_EM_ CTLs compared to the one containing only naïve/T_CM_ when assessed *ex vivo*.

In addition to its variable expression between memory subsets, our previous analysis indicated that Tpl2 mRNA was significantly induced by IL-12 in during primary expansion in response to TCR engagement [8]. This observation was confirmed by quantifying both total mRNA and Tpl2 protein in human CD8^+^ T cells 3.5 days post-stimulation (Figure 1E, F). IL-12, but not IFN-α, was able to significantly up-regulate the expression of Tpl2 mRNA in CTLs in vitro when compared to the neutralized condition (Figure 1E). The same trend was observed at the protein level where IL-12 alone significantly up regulated Tpl2 protein (Figure 1F). Thus, IL-12 activation significantly induces expression of Tpl2 in human CD8^+^ T cells.

### Tpl2 is dispensable for pro-inflammatory cytokine secretion in murine CTLs

**Figure 2:**
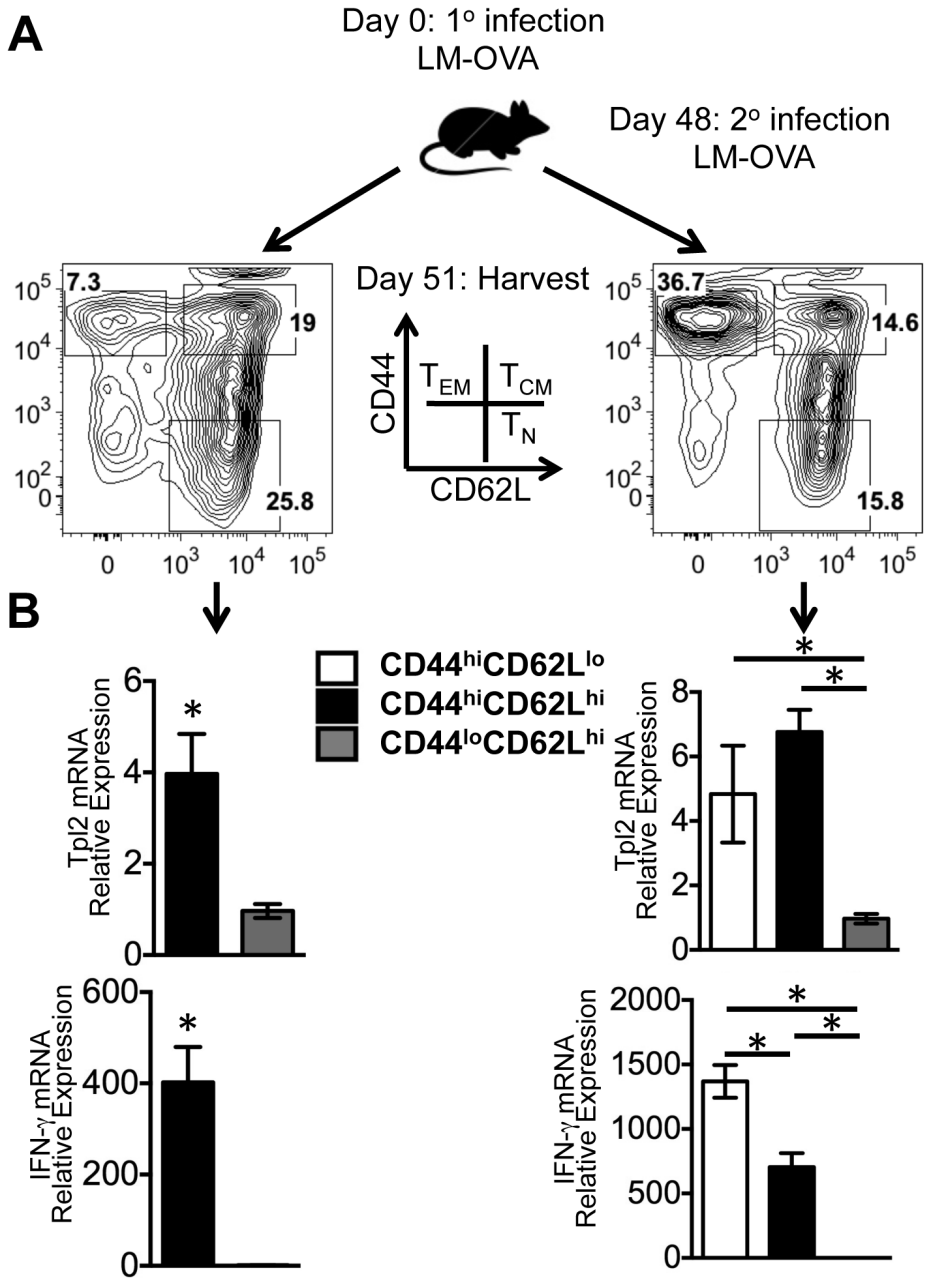
Differential Tpl2 mRNA expression in murine naïve versus effector/memory CTL subpopulations following *Listeria monocytogenes* infection. (A) WT C57BL/6 mice were infected intravenously with 2000 CFU of LM-OVA. One cohort of the mice was re-infected with 20,000 CFU of LM-OVA 48 days post infection. Spleen and LN from all mice were collected 51 days post 1° infection and surface receptor staining was performed for CD8, CD44, and CD62L. T_EM_, T_CM_, and T_N_ CTLs were defined as indicated for sorting and total RNA was isolated immediately after sorting. The cells were pooled from the mice under the same treatment group. (B) Tpl2 and IFN-γ mRNA were measured by qRT-PCR within the CD44^hi^CD62L^lo^ (white bar, only from the mice with both 1° and 2° infections due to limiting cell numbers), CD44^hi^CD62L^hi^ (black bar), and CD44^l^°CD62L^hi^ (gray bar) sorted CTLs, and relative expression was determined by comparing to CD44^l^°CD62L^hi^ population from each infection group. PPIA was used as the reference gene. This experiment was performed twice with 3–5 animals/group.

In order to test the role of Tpl2 in CTL effector functions *in vivo*, we first established the expression pattern of Tpl2 in murine CD8^+^ CTLs. In order to generate effector and memory CD8^+^ T cells *in vivo*, WT C57BL/6 mice were infected intravenously with the intracellular bacteria *L. monocytogenes* as a model infection (Figure 2A). In order to distinguish subpopulations of naïve, effector and memory populations, we utilized the two widely used cell surface markers, CD44 and CD62L, to delineate the T_EM_, T_CM_, and T_N_ CTLs from mice with resting memory as well as from mice re-challenged with LM-OVA. There was insufficient number of CD44^hi^CD62L^lo^ cells sorted from the mice that received 1° infection only and thus not enough RNA to use in the qRT-PCR. Otherwise, following isolation of each population, qRT-PCR analysis revealed that Tpl2 was expressed at a higher level in both T_CM_ and T_EM_ CTLs compared to T_N_ CTLs (Figure 2B, top panels). Furthermore, Tpl2 expression pattern correlated with expression pattern of IFN-γ (Figure 2B, bottom panels). Thus, in response to LM-OVA, Tpl2 mRNA expression was higher in effector and memory CTLs in mice. Thus, the endogenous expression of Tpl2 in T_EM_ in murine cells paralleled our observations in human T_EM_ cells.

**Figure 3:**
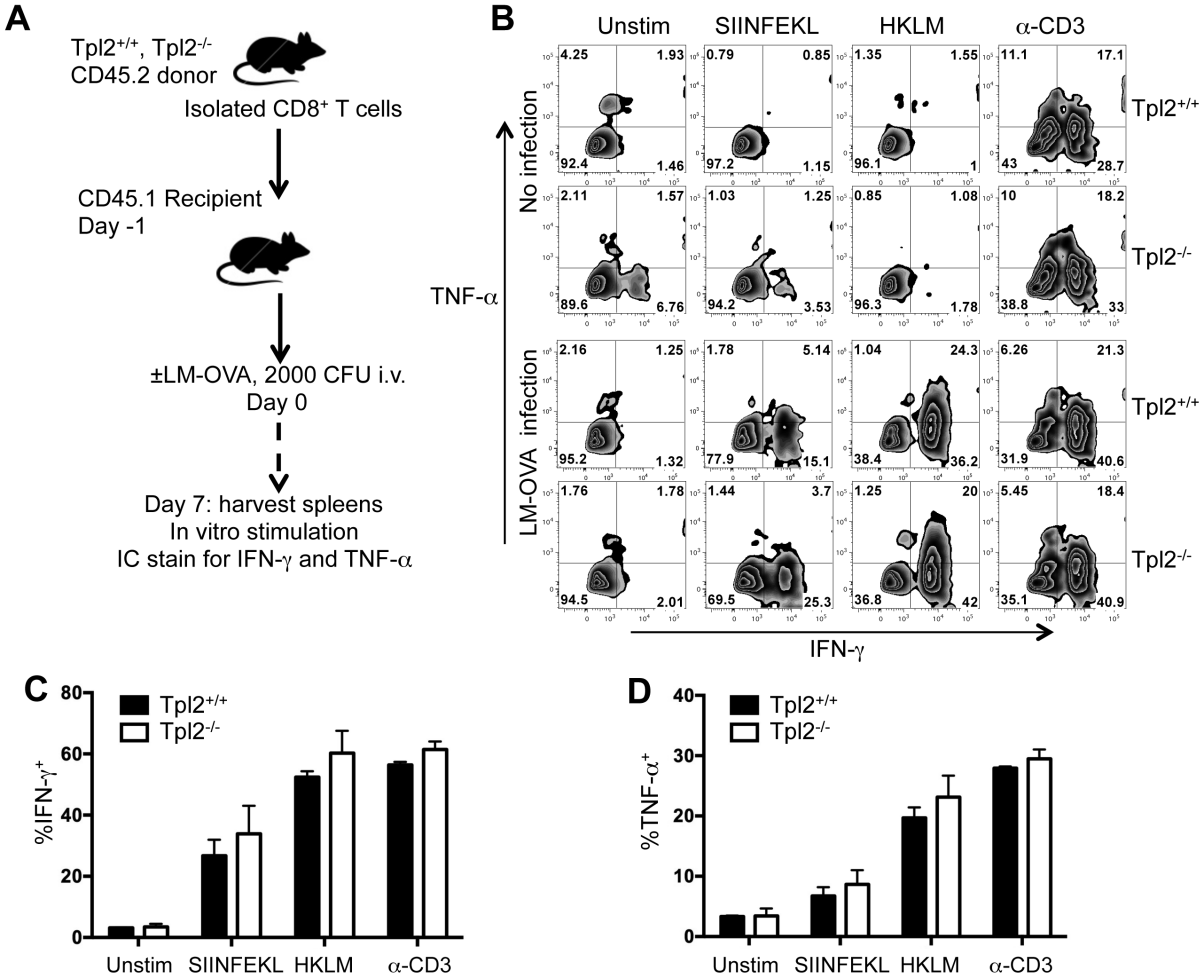
*L. monocytogenes infection primes cytokine expression in Tpl2-defecient CTLs*. (A) Total CD8^+^ T cells were isolated from Tpl2^+/+^ (n = 4) and Tpl2^−/−^ (n = 4) mice, which carried the CD45.2 congenic marker. Isolated cells from each mouse were separately injected into three CD45.1 recipient mice at 1e^6^ cells per mouse, intravenously on day -1. On day 0 mice were either infected with 2000 CFU of LM-OVA or left uninfected. Spleens were harvested day 7 post infection and splenocytes were cultured with SIINFEKL (10 nM), HKLM (5 mg/mL), soluble anti-CD3 (0.5 μg/mL), or left unstimulated for 18 h. Monensin was added to the cultures for the last 6 h. Intracellular expression of IFN-γ and TNF-α were measured by flow cytometry along with the surface markers CD8a and CD45.2. (B) Intracellular IFN-γ (x-axis) and TNF-α (y-axis) expression after 2° stimulation is represented in dot plots for both Tpl2^+/+^ and Tpl2^−/−^ mice. Transferred cells were identified based on positive staining for CD8α and CD45.2 surface marker. (C–D) Total IFN-γ (C) and TNF-α (D) expressing cells were quantified as a percentage of the identified total transferred (CD8^+^CD45.2^+^) T cells. Mean±SEM is plotted representing triplicate donor mice with Tpl2^+/+^ in black and Tpl2^−/−^ in white bars. No statistically significant differences in cytokine expression were observed between strains as assessed by two-way ANOVA.

To determine the role of Tpl2 in regulating CTL effector responses *in vivo*, we utilized the Tpl2^−/−^ strain [13]. First, no gross differences were observed between the Tpl2^+/+^, Tpl2^+/−^, and Tpl2^−/−^ mice comparing the proportions of CD4 and CD8 expression patterns in thymus, blood, LN, and spleen of uninfected naïve animals (Figure S1 in File S1). This is in agreement with previous reports demonstrating normal ratios of immune cells and without any obvious phenotype [13], [21]. We wished to determine if Tpl2^−/−^ CD8^+^ T cells were capable of expressing effector cytokines when developing in response to a pathogen *in vivo*. Isolated CD8^+^ T cells from both Tpl2^+/+^ and Tpl2^−/−^ mice were adoptively transferred into CD45.1 recipient mice (Figure 3A). Recipient animals were then infected with LM-OVA and allowed to recover. Splenocytes were harvested at d7 post infection and stimulated in the presence of SIINFEKL peptide, HKLM, or anti-CD3. Using CD45.2 as a marker for the donor cells we detected antigen-specific IFN-γ and TNF-α expression by intracellular staining only from the mice that were infected with LM-OVA (Figure 3B). However, no statistically significant differences were observed in either IFN-γFigure 3C) or TNF-α (Figure 3D) expression between wild type and Tpl2^−/−^ CD8^+^ T cells under any of the re-stimulation conditions.

**Figure 4:**
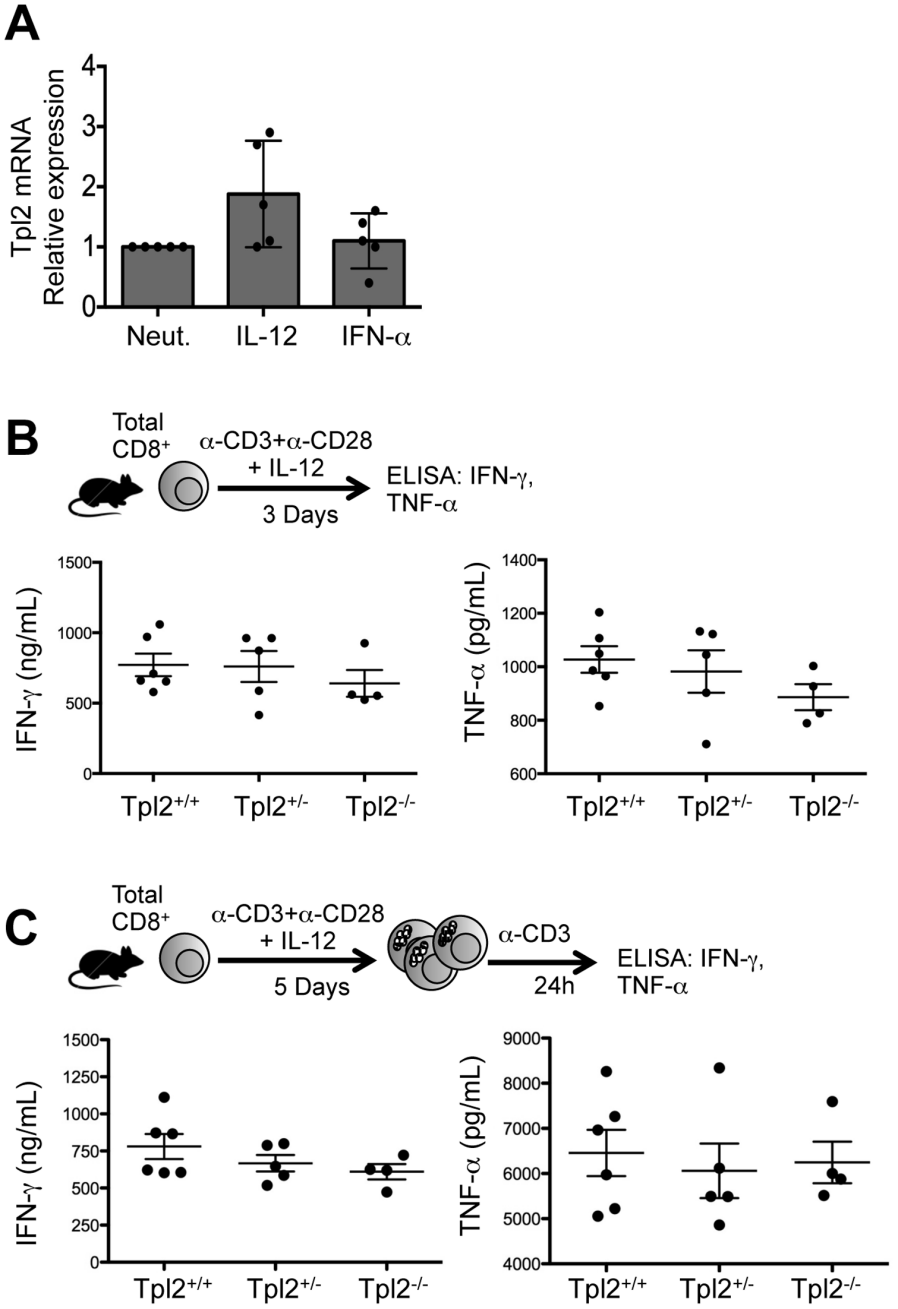
Tpl2 expression is dispensable for effector cytokine secretion from murine CTLs. (A) Total CD8^+^ T cells from C57BL/6 WT murine splenocytes were isolated using negative isolation kit and stimulated *in vitro* with plate-bound anti-CD3+anti-CD28 under defined cytokine conditions (Neutralized, IL-12, and IFN-α). Relative expression of Tpl2 mRNA was measured by qRT-PCR. and determined for each mouse separately compared its own neutralized condition. (B) Total CD8^+^ T cells were isolated from spleens and lymph nodes of Tpl2^+/+^ (n = 6), Tpl2^+/−^ (n = 5), and Tpl2^−/−^ (n = 4) mice using negative isolation kit. Cells were stimulated *in vitro* with plate-bound anti-CD3 and anti-CD28 in the presence of IL-12 for 3 days. IFN-γ (left panel) and TNF-α (right panel) in the supernatant were measured by ELISA. Each individual data point represents a separate mouse. (C) Purified CD8^+^ T cells from Tpl2^+/+^ (n = 6), Tpl2^+/−^ (n = 5), and Tpl2^−/−^ (n = 4) mice were cultured for 3 days as in (A), and split 1:10 with 50 U/mL rhIL-2. On day 5 post stimulation, cells were re-stimulated with plate-bound anti-CD3+rhIL-2 for 24 h. IFN-γ (left panel) and TNF-α (right panel) were measured by ELISA. Individual dot represents a separate mouse used in this experiment and is representative of four individual experiments. No statistically significant differences in cytokine expression were observed between strains as assessed by two-way ANOVA.

Next, we tested whether Tpl2 was necessary for IL-12-induced effector CTL function *in vitro*. Although, IL-12 was able to drive murine effector CTL differentiation, there was no statistically significant regulation of Tpl2 by IL-12 in murine CTLs *in vitro* (Figure 4A). This is consistent with previous data from Mescher and colleagues [4]. We further tested the ability of WT and Tpl2^−/−^ CD8^+^ T cells to differentiate into primary effector cells in vitro in response to IL-12 priming conditions. Consistent with their ability to develop into primary effectors in vivo (Figure 3) we found that CTLs from Tpl2^+/+^, Tpl2^+/−^, and Tpl2^−/−^ mice were equally competent in their ability to secrete either IFN-γor TNF-α in response to IL-12 in vitro (Figure 4B). Moreover, no differences in IFN-γ or TNF-α secretion were observed upon secondary stimulation of IL-12-polarized murine CTLs *in vitro* (Figure 4C). Thus, Tpl2 was not required for effector cytokine expression and secretion in murine CTLs.

### Pharmacological inhibition of Tpl2 blocks effector cytokine secretion from human but not murine CTLs *in vitro*

**Figure 5:**
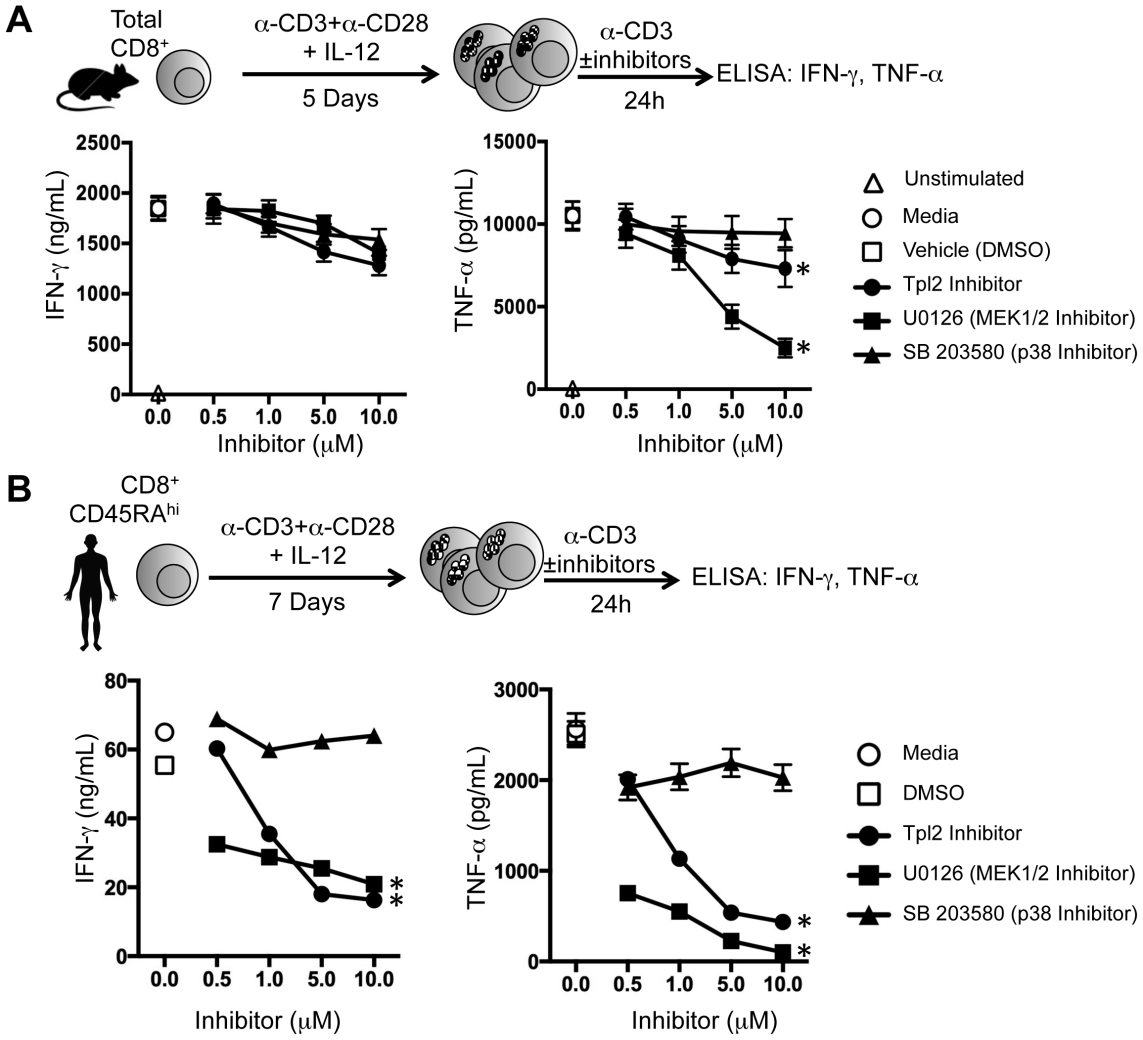
Pharmacological inhibition of Tpl2 blocks effector cytokine secretion from human but not murine CTLs *in vitro*. (A) Total CD8^+^ T cells were isolated from WT C57BL/6 splenocytes and stimulated with plate-bound anti-CD3+anti-CD28 in the presence of rhIL-2 and rmIL-12. On day 3 post stimulation, the cells were split 1:10 in cIMDM+50 U/mL rhIL-2. On day 5 post stimulation, cells were harvested, counted, and re-stimulated for 24 h with plate-bound anti-CD3+50 U/mL rhIL-2 in the presence or absence of the indicated inhibitor conditions to test for effector cytokines secretion. IFN-γ (left panel) and TNF-α (right panel) in the supernatant were measured using ELISA. Mean±SEM is plotted and the error represents five individual mice, n = 5. (B) Naïve CD8^+^ (CD8^+^CD45RA^+^) T cells were isolated by negative selection or FACS from healthy human PBMC and stimulated with plate-bound anti-CD3+anti-CD28 and rhIL-12. Cells were split 1:10 with 100 U/mL IL-2 on d3 post stimulation. On d7, cells were re-stimulated with plate-bound anti-CD3+rhIL-2 (200 U/mL)±indicated kinase inhibitors. Supernatant was collected 24 h post 2^o^ stimulation. IFN-γ (left panel) and TNF-α (right panel) in the supernatant were measured by ELISA. Data shown are representative of five independent experiments and mean±SEM is plotted. *, p≤0.05 compared to control treated groups.

Given that Tpl2^−/−^ CTLs were not defective in effector cytokine secretion, we wished to determine if Tpl2 was necessary for effector cytokine secretion from human CTLs *in vitro*. Since the human and murine Tpl2 gene products are 93% identical at the amino acid level [22], we utilized a commercially available small molecule inhibitor of Tpl2 function (Tpl2 inhibitor) to test the role of Tpl2 in regulating cytokine secretion in CD8^+^ T cells from both species. This inhibitor is cell permeable, reversible, and blocks Tpl2 activation in an ATP-dependent manner [23], [24]. Further, this Tpl2 inhibitor has been shown to block TNF-α secretion from murine bone marrow derived macrophages, as well as human PBMCs responding to LPS stimulation [24]–[26]. Thus, the commercially available Tpl2 inhibitor can block Tpl2 activation in both mouse and human innate immune cells. In order to contrast the effects of this inhibitor with other well-characterized inhibitors of MAP kinases, we used commercially available small molecule inhibitors of ERK and p38. U0126 blocks activation of MEK1/2, which phosphorylates ERK, and SB 203580 directly blocks p38 activation. First, murine WT C57BL/6 CD8^+^ T cells were cultured *in vitro* in the presence of IL-12 and then re-stimulated with plate-bound anti-CD3 alone in the presence of the kinase inhibitors to block activation of Tpl2, MEK1/2, or p38 (Figure 5A). Consistent with our findings from the murine Tpl2^−/−^ cells, the Tpl2 inhibitor did not alter IFN-γ secretion compared to either the MEK or p38 inhibitor. While significantly different from the MEK inhibitor, the Tpl2 inhibitor reduced TNF-α secretion by 10% compared to the p38 inhibitor. U0126 was able to significantly block TNF-α secretion, but not IFN-γ secretion (Figure 5A). Taken together, both the analysis of Tpl2^−/−^ CTLs and pharmacological studies demonstrated that Tpl2 function was not required for effector cytokine secretion in murine CTLs.

We previously demonstrated that IL-12, but not IFN-α, regulated effector function in human CD8^+^ T cells *in vitro* as measured by IFN-γ and TNF-α secretion [7]. Given the robust induction of Tpl2 by IL-12 in humαn, but not mouse CD8^+^ T cells, we wished to test whether Tpl2 was necessary for effector cytokine secretion from IL-12 programmed human CTLs. Tpl2 inhibitor treatment significantly reduced both IFN-γ and TNF-α secretion from IL-12 polarized CTLs in a dose-dependent manner (Figure 5B). Moreover, significant reduction in IFN-γ and TNF-α secretion was observed when activation of MEK1/2 was blocked using U0126 (Figure 5B). As expected [27], the p38 inhibitor (SB 203580) did not alter TCR-induced IFN-γ or TNF-α secretion from IL-12 programmed human CTLs (Figure 5B). As controls for activation, we found that the percentage of cells expressing the activation marker CD25 did not change with the treatment of any of the inhibitors for 24 h during the secondary stimulation (Figure S2 in File S1). Together, these data suggested that TCR-mediated activation of IL-12 programmed human effector CTLs *in vitro* leads to IFN-γ and TNF-α secretion, and the Tpl2 inhibitor suppressed this process.

### Tpl2 and MEK activation regulate human T_EM_ CTL effector function

**Figure 6:**
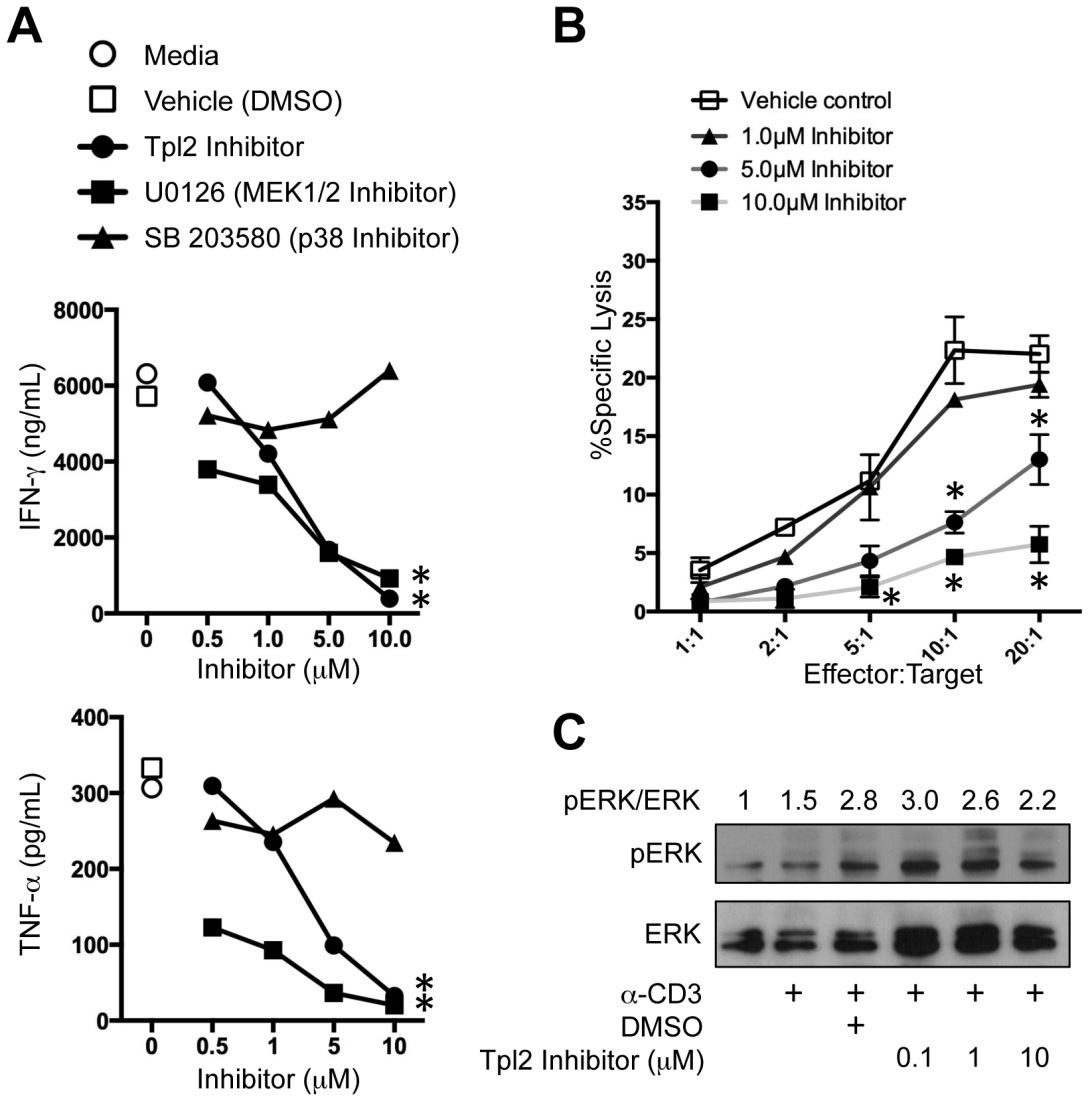
Pharmacological blockade of Tpl2 significantly impairs human CTL cytokine secretion and lytic activity. CD8^+^CCR7^lo^ T_EM_ CTLs were sorted from healthy human PBMCs. (A) Sorted cells were stimulated with plate-bound anti-CD3 and rhIL-2 (200 U/mL)±indicated kinase inhibitors. Supernatants were collected at 24 h post stimulation. IFN-γ (top panel) and TNF-α (bottom panel) in the supernatant were measured using ELISA and mean±SEM is plotted for a representative of five similar experiments. (B) Sorted CD8^+^CCR7^lo^ T_EM_ CTLs were used in redirected lysis assay at the indicated Effector:Target ratios using anti-CD3 coated THP-1 target cells±Tpl2 inhibitor at the indicated concentrations. Killing of target cells is plotted as percent specific lysis, mean±SD plotted. Data shown are representative of two similar experiments from separate healthy adult donors. (C) Purified human CD8^+^ T cells were expanded for 5 days on anti-CD3/anti-CD28-coated plates in the presence of rhIL-12. Cells were rested for 24 h then restimulated with biotin-anti-CD8 + biotin-anti-CD3 with crosslinking streptavidin for 10 min in the absence or presence of the Tpl2 inhibitor at the indicated concentrations. Cell lysates were prepared and assessed for p-ERK and ERK by western blotting. Data are representative of 3 separate experiments.

Human T_EM_ CTLs purified from peripheral blood retain the ability to exert effector functions immediately upon TCR stimulation (Figure 1A–C) and express higher levels of Tpl2 mRNA compared to T_N_ CTLs (Figure 1D). Using the small molecule inhibitors, we tested whether Tpl2 activation is necessary for cytokine secretion and lytic activity of human T_EM_ cells *ex vivo*. Both IFN-γ and TNF-α secretion were significantly reduced in a dose-dependent manner when CD8^+^CCR7^lo^ sorted CTLs were stimulated in the presence of the Tpl2 inhibitor (Figure 6A). When cytolytic activity of T_EM_ CTLs was tested *in vitro*, the Tpl2 inhibitor markedly reduced their ability to kill target cells in a dose dependent manner (Figure 6B). Further, we also observed a dose-dependent reduction in IFN-γ and TNF-α secretion from T_EM_ CTLs in the presence of U0126, but not SB 203580 (Figure 6A). CTL viability remained unchanged with the treatment of 10 μM Tpl2 inhibitor as assessed by annexinV/7AAD staining when compared with TCR stimulation alone (Figure S3 in File S1). Together, these data suggested that TCR mediated activation of human T_EM_ CTL effector functions may be Tpl2-MEK-ERK dependent but p38 independent. To test this directly, we assessed TCR-mediated ERK phosphorylation in human T_EM_ in the absence or presence of the Tpl2 inhibitor. TCR activation via anti-CD3 induced a 1.5-fold increase in p-ERK, which was enhanced by the DMSO vehicle control (Figure 6C). Although the p-ERK/ERK ratio was reduced by approximately 25% at the highest concentration of the Tpl2 inhibitor, this result indicates that the block in TCR-mediate cytokine secretion may be due to alternate downstream targets of Tpl2.

## Discussion

The role of Tpl2 in innate immune cells such as dendritic cells and macrophages in mice is well documented. Both the Tpl2 knockout mouse model as well as the use of pharmacological inhibitors have demonstrated an essential role for Tpl2 in TNF-α production from murine dendritic cells and macrophages [12], [13], [28]. Although it has been shown that Tpl2 deficiency renders Th1 cells inadequate at IFN-γ secretion, no studies have interrogated the role of Tpl2 in CTL effector functions [15]. This is perhaps due to the fact that when the TCR2C transgenic mice were crossed with the Tpl2^−/−^, the transgenic CD8^+^ T cells were transformed into chronically stimulated T cells and developed into lymphomas, which led the authors to conclude that Tpl2 may be playing an anti-proliferative role in murine CTLs [29]. The functional consequences of TPL2 deletion in TCR2C transgenic CTLs was not tested, and it remains inconclusive if Tpl2 plays any role in effector function from CD8^+^ T cells. In this study, we sought to answer this question using both human and murine CD8^+^ T cells.

The innate inflammatory cytokine IL-12 plays a significant role in programming human effector CTL function [7], [8]. Along with along with pro-inflammatory cytokines (IFNG, TNF, IL-8, etc), IL-12 also induces expression of the MAP kinase pathway intermediate Tpl2/MAP3K8. In this study, we found that human T_EM_ CTLs expressed higher levels of MAP3K8 *ex vivo* when compared to T_N_+T_CM_ CTLs (Figure 1D). We further demonstrated that IL-12 induced the expression of Tpl2 mRNA and protein in human CD8^+^ T cells, suggesting that Tpl2 may play a critical role in their function. Indeed, blocking the enzymatic activity of Tpl2 kinase with a specific small molecule inhibitor significantly reduced cytokine secretion and cytotoxicity. Additionally, we found that the MEK1/2 inhibitor also blocked effector function in human CTLs, indicating the involvement of MEK-ERK pathway. These data support a unique role for Tpl2 pathway in regulating TCR-mediated acute effector cytokine secretion and lytic activity. Interestingly, we observed only a modest reduction of ERK phosphorylation in the presence of the Tpl2 inhibitor, which suggests that the alternative downstream targets of Tpl2 may synergize with ERK activation to drive full effector function in memory cells. During the transition from naïve to effector/TEM, CD8^+^ T cells down regulate CD28 and become uncoupled from their need for costimulation during recall antigen responses. It is possible that Tpl2 plays a role in substituting for components of co-stimulation that are lost during that transition. The downstream targets of Tpl2 activation in this scenario are under further investigation.

In contrast to human cells, Tpl2 was completely dispensable for CTL function and memory cell development in mice. Further, we found no significant regulation of Tpl2 by IL-12 in murine CD8^+^ T cells, as was found by Watford and colleagues in CD4^+^ T cells [15]. However, the lack of Tpl2 in innate immune cells has been shown to reduce TNF-α secretion among other phenotypes [9], [12]. There are several possible explanations for the differential requirement of Tpl2 in murine CTL activation. First, there are at least 16 other MAPKKK that have been identified to have the dual-specificity kinase activity upstream of the major MAP kinase pathways [30]. It is possible that any of these MAPKKK may be playing a compensatory role in activation of murine CTLs in the absence of Tpl2. Furthermore, blocking ERK activation in murine CTLs through MEK1/2 inhibition blocks TNF-α secretion but pharmacological inhibition of Tpl2 has no effect. Thus, we can conclude that there is likely a Tpl2 independent mechanism of ERK activation leading to effector cytokine secretion in murine CTLs.

Inflammation develops as a cascade effect of chemokine-regulated cellular recruitment followed by innate and adaptive mechanisms of pro-inflammatory cytokine secretion. The state of inflammation is usually resolved upon pathogen clearance and tissue repair. However, chronic inflammation can have debilitating effects in a variety of autoimmune disorders, such a rheumatoid arthritis. Although blockade of TNF-α secretion with biologicals profoundly improves treatment outcomes of these diseases [31], there has been accelerated interest in developing small molecule inhibitors of these pathways. Many of these inhibitors were developed to target protein kinases since the discovery of their ability to block not only the relevant kinase activity but also the production of pro-inflammatory cytokines such as IL-1β and TNF-α[32]. Recently, Tpl2 has been identified as a ‘druggable’ target due to its role in activating innate immune cells to produce TNF-α in mice and human [23], [24], [33]. Therapeutic use of Tpl2 inhibitors has been suggested to impede prolonged and uncontrolled inflammation in diseases such as rheumatoid arthritis and inflammatory bowel disease based on preliminary studies in mice [26], [34]–[36]. In the present study, we have shown that human CTL function is drastically reduced when the cells are stimulated in the presence of Tpl2 inhibitor. While the downstream targets of this inhibition are currently unknown, this pathway is specific to human cells as mouse cells were resistant to the effects of the inhibitor. Consequently, future studies must be directed to understanding how this pathway regulates CTL function in a species-specific manner.

In summary, this study has shown that human CTL effector function may be regulated by the IL-12 induced serine-threonine protein kinase Tpl2, likely upstream of MEK/ERK mediated pathway. We have discovered a species-specific role for Tpl2 in regulating effector function of human, but not murine CTL. Blocking Tpl2 function upstream of MEK-ERK activation markedly diminished effector cytokine secretion and cytolytic capabilities of human effector and T_EM_ CTLs. The effects of blocking the different MAP kinases can have different consequences in a cell type dependent manner. Thus, any therapeutic use of kinase inhibitors must be considered carefully by weighing out the cost-benefit ratio of the outcome on immune fitness.

## Supporting Information

File S1 **File S1 contains the following: Figure S1. Tpl2^−/−^ and Tpl2^+/+^ mice display comparable CD4 and CD8 profiles in primary and secondary lymphoid organs.** Thymus, peripheral blood, lymph nodes, and spleen were harvested from 4 month old Tpl2^+/+^, Tpl2^+/−^, and Tpl2^−/−^ mice. Cells were stained for surface expression of CD4 and CD8 *ex vivo* and analyzed by flow cytometry. **Figure S2. TCR-mediated induction of CD25 in human CD8+ T cells is not altered by specific MAP kinase inhibitors.** Naïve CD8^+^ (CD8^+^CD45RA^+^) T cells were isolated by negative selection from healthy human PBMCs and stimulated with plate-bound anti-CD3+anti-CD28 and rhIL-12. Cells were split 1:10 with 100 U/mL IL-2 and cultured until day 7 when cells were counted and re-stimulated with plate-bound anti-CD3±indicated inhibitors or left unstimulated. CD25 expression was measured by staining for the surface marker and analysis with flow cytometry 24 h post 2° stimulation. Data shown are representative of 3 experiments from separate healthy donors. Percent of CD25^+^ cells within the live gate were determined and mean±SD plotted. **Figure S3. Specific MAP kinase inhibitors do not alter human T_EM_ CTL cell viability.** CD8^+^CCR7^lo^ T cells were isolated from healthy human PBMCs by FACS sorting. Cells were stimulated with plate-bound anti-CD3 in the presence of the highest concentration of the Tpl2 inhibitor (10 mM) used throughout the study. Cell viability was measured 24 h post stimulation by staining for AnnexinV and 7AAD. Data are expressed as dot plots of total events without live cell gating.
(DOCX)
