# Supplementary material for: Pharmacological Inhibition of TPL2/MAP3K8 Blocks Human Cytotoxic T Lymphocyte Effector Functions
Source: PLoS One. 2014 Mar 18;9(3):e92187. doi: 10.1371/journal.pone.0092187 (PMC3958505; doi:10.1371/journal.pone.0092187)
Supplement: File S1 — contains the following: Figure S1. Tpl2−/− and Tpl2+/+ mice display comparable CD4 and CD8 profiles in primary and secondary lymphoid organs. Thymus, peripheral blood, lymph nodes, and spleen were harvested from 4 month old Tpl2+/+, Tpl2+/−, and Tpl2−/− mice. Cells were stained for surface expression of CD4 and CD8 ex vivo and analyzed by flow cytometry. Figure S2. TCR-mediated induction of CD25 in human CD8+ T cells is not altered by specific MAP kinase inhibitors. Naïve CD8+ (CD8+CD45RA+) T cells were isolated by negative selection from healthy human PBMCs and stimulated with plate-bound anti-CD3+anti-CD28 and rhIL-12. Cells were split 1:10 with 100 U/mL IL-2 and cultured until day 7 when cells were counted and re-stimulated with plate-bound anti-CD3±indicated inhibitors or left unstimulated. CD25 expression was measured by staining for the surface marker and analysis with flow cytometry 24 h post 2° stimulation. Data shown are representative of 3 experiments from separate healthy donors. Percent of CD25+ cells within the live gate were determined and mean±SD plotted. Figure S3. Specific MAP kinase inhibitors do not alter human TEM CTL cell viability. CD8+CCR7lo T cells were isolated from healthy human PBMCs by FACS sorting. Cells were stimulated with plate-bound anti-CD3 in the presence of the highest concentration of the Tpl2 inhibitor (10 mM) used throughout the study. Cell viability was measured 24 h post stimulation by staining for AnnexinV and 7AAD. Data are expressed as dot plots of total events without live cell gating. (DOCX) [file pone.0092187.s001.docx]

Supplemental Figure 1 (Figure S1)

**Figure S1. Tpl2^-/-^ and Tpl2^+/+^ mice display comparable CD4 and CD8 profiles in primary and secondary lymphoid organs.**

Thymus, peripheral blood, lymph nodes, and spleen were harvested from 4 month old Tpl2^+/+^, Tpl2^+/-^, and Tpl2^-/-^ mice. Cells were stained for surface expression of CD4 and CD8 *ex vivo* and analyzed by flow cytometry.

Supplemental Figure 2 (Figure S2)

**Figure S2.** **TCR-mediated induction of CD25 in human CD8+ T cells is not altered by specific MAP kinase inhibitors**.

Naïve CD8^+^ (CD8^+^CD45RA^+^) T cells were isolated by negative selection from healthy human PBMCs and stimulated with plate-bound anti-CD3+anti-CD28 and rhIL-12. Cells were split 1:10 with 100U/mL IL-2 and cultured until day 7 when cells were counted and re-stimulated with plate-bound anti-CD3±indicated inhibitors or left unstimulated. CD25 expression was measured by staining for the surface marker and analysis with flow cytometry 24h post 2^o^ stimulation. Data shown are representative of 3 experiments from separate healthy donors. Percent of CD25^+^ cells within the live gate were determined and mean±SD plotted.

Supplemental Figure 3 (Figure S3)

**Figure S3. Specific MAP kinase inhibitors do not alter human T_EM_ CTL cell viability.**

CD8^+^CCR7^lo^ T cells were isolated from healthy human PBMCs by FACS sorting. Cells were stimulated with plate-bound anti-CD3 in the presence of the highest concentration of the Tpl2 inhibitor (10mM) used throughout the study. Cell viability was measured 24h post stimulation by staining for AnnexinV and 7AAD. Data are expressed as dot plots of total events without live cell gating.
